# Pulmonary Sarcomatoid Carcinoma: What Makes This Rare Lung Cancer So Challenging?

**DOI:** 10.7759/cureus.63045

**Published:** 2024-06-24

**Authors:** Badr Kharouaa, Amine Hayoune, Sara Gartini, Meriem Rhazari, Afaf Thouil, Hatim Kouismi

**Affiliations:** 1 Department of Respiratory Diseases, Faculty of Medicine and Pharmacy of Oujda, Mohammed VI University Hospital, Mohammed First University, Oujda, MAR; 2 Department of Respiratory Diseases, Research and Medical Sciences Laboratory, Faculty of Medicine and Pharmacy of Oujda, Mohammed VI University Hospital, Mohammed First University, Oujda, MAR

**Keywords:** lung carcinoma, smoking, hemiplegia, sarcoma, pulmonary sarcoma

## Abstract

Pulmonary sarcomatoid carcinoma (PSC) is a rare and aggressive subtype of non-small cell lung carcinoma (NSCLC). This case report describes a 55-year-old male with a significant smoking history who initially presented with left hemiplegia. Imaging studies revealed brain metastases and a spiculated parenchymal lung nodule in the left apical region. Histopathological examination confirmed PSC through a CT-guided biopsy. The patient's condition rapidly deteriorated, leading to death before the initiation of planned palliative chemotherapy. This report highlights the diagnostic challenges and poor prognosis associated with PSC, emphasizing the need for further research into effective treatment strategies.

## Introduction

Lung cancer stands as the primary cause of cancer-related fatalities worldwide, marked by its aggressive nature and grim prognosis [1,2]. It is categorized into two principal types: small cell lung cancer, comprising 20% of cases and recognized for its high malignancy, and non-small cell lung cancer, encompassing the remaining 80% [3,4].

Pulmonary sarcomatoid carcinoma (PSC) represents an exceedingly rare subset of lung cancers, constituting a mere 0.1% to 0.4% of all malignant lung tumors [3]. Predominantly affecting older males with extensive smoking histories, PSC predominantly manifests in the upper lung lobes [5,6,7]. The clinical presentation of PSC lacks specificity, often manifesting with nonspecific symptoms like dyspnea, cough, hemoptysis, chest pain, and weight loss [8,9,10].

Due to its aggressive nature and proclivity for frequent metastasis, PSC exhibits limited response rates to conventional treatments such as chemotherapy, radiotherapy, and neoadjuvant chemotherapy. However, recent advancements in gene sequencing, targeted therapies, and immunotherapies have shown significant promise in the treatment of various malignancies. The advent of next-generation sequencing (NGS) has enabled the identification of specific genetic mutations and alterations, facilitating the development of personalized treatment plans tailored to the molecular profile of individual tumors [3]. Targeted therapies, such as tyrosine kinase inhibitors and monoclonal antibodies, have demonstrated efficacy in targeting specific oncogenic pathways, thereby improving treatment outcomes and reducing adverse effects. Additionally, the field of immunotherapy has revolutionized cancer treatment, with immune checkpoint inhibitors such as pembrolizumab and nivolumab showing durable responses in various cancer types by enhancing the body's immune response against tumor cells. These advancements underscore the importance of integrating molecular diagnostics and novel therapeutic approaches in the management of cancer, paving the way for more effective and individualized treatment strategies [3].

PSC is a rare and aggressive lung cancer, constituting 0.1% to 0.4% of cases. Lung cancer causes 1.8 million deaths annually. A study by Lin Yongbin et al. found PSC mainly in young male smokers, with a median survival of 19.1 months and a five-year survival rate of 17.4%. Factors for better survival include no distant metastasis, a higher body mass index (BMI), normal hemoglobin, smaller tumors, and complete resection. Prognostic factors are M stage, pathology, and complete resection [8].

Given its rarity, rapid progression, limited survival, and heterogeneous pathological features, formulating therapeutic strategies for PSC has posed significant challenges [2]. This study presents a case of PSC and contributes valuable insights to the medical literature.

## Case presentation

A 55-year-old patient with a 40-pack-year smoking history was initially admitted to the emergency department for the management of left hemiplegia. The patient's vital signs were normal. Physical examination revealed a positive pyramidal syndrome on the left side of the body and wheezing and rales at the apices. The remainder of the examination was unremarkable.

**Figure 1:**
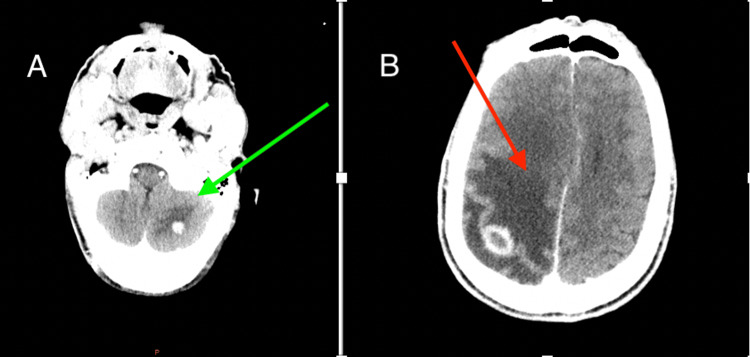
CT brain scan A: cortico-subcortical hypodense area indicates perilesional edema with a mass effect on the ipsilateral ventricle and a 9-mm midline shift to the left (green arrow); B: intra-axial lesions in the supra- and subtentorial regions; the largest lesion is located in the right parietal region, suggesting secondary lesions (red arrow).

A brain CT scan revealed intra-axial lesions in both supra- and subtentorial regions. These lesions were rounded, irregularly contoured, and exhibited intense enhancement with peripheral enhancement after contrast injection. The largest lesion, measuring 20x18 mm, was located in the right parietal region, suggesting metastasis. Additionally, a cortico-subcortical hypodense area indicative of perilesional edema caused a mass effect on the ipsilateral ventricle, along with a 9 mm deviation of the midline structures to the left (Figure 1).

**Figure 2:**
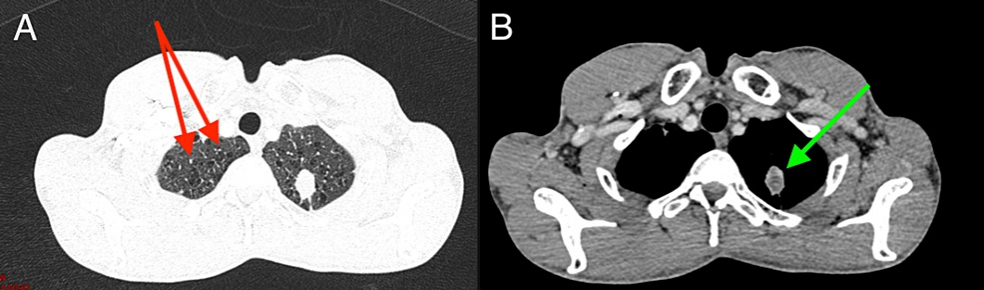
Axial chest CT scan A: lung window; B: mediastinal window demonstrating spiculated parenchymal lung nodules in the left apical region (green arrow), with bilateral pulmonary parenchymal nodules and micronodules (red arrows), with the largest measuring 9 mm in the sub-pleural middle lobar fissure and 8 mm in the sub-pleural right lower lobe.

A thoraco-abdomino-pelvic CT scan identified a spiculated parenchymal lung nodule in the left apical region measuring 22x16 mm, extending to 23 mm. This nodule was closely associated with the parietal pleura near the posterior arch of the second left rib, without adjacent bone lysis (Figure 2). Bilateral pulmonary parenchymal nodules and micronodules were also observed, with the largest measuring 9 mm in the subpleural middle lobar fissure and 8 mm in the subpleural right lower lobe.

Additionally, a rounded, hypodense lesion in the pancreatic body with heterogeneous enhancement post-contrast injection, as well as nodular lesions in the body and inner arm of the left adrenal gland with a spontaneous density > 10 HU, were identified but not further explored due to the patient's refusal.

**Figure 3:**
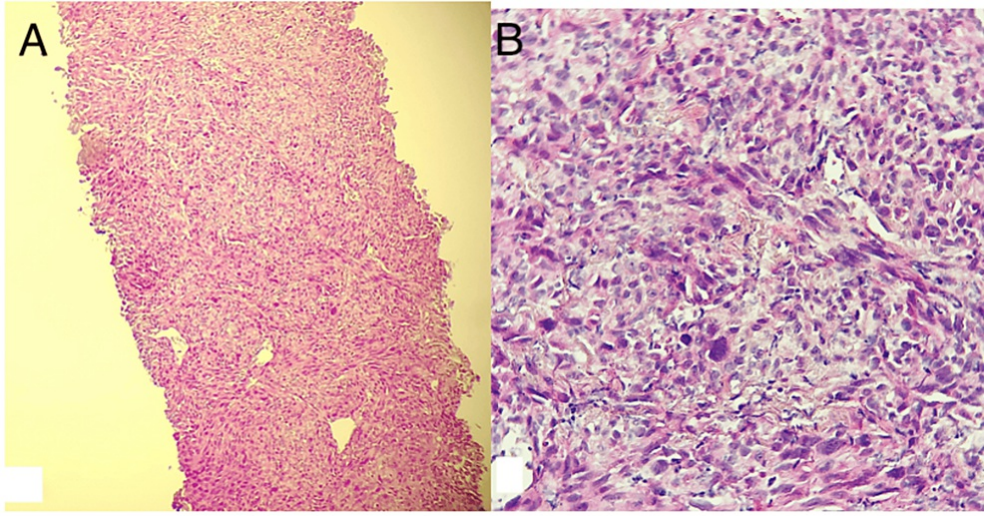
Histological examination of the bronchobiopsy A: tumor proliferation arranged in diffuse sheets (hematoxylin and eosin (H&E), 100X); (B): cells that are sometimes cohesive, sometimes spindle-shaped, atypical, hyperchromatic, anisokaryosis, and focally mitotic (H&E, 400X).

A bronchial fibroscopy revealed no pathological processes or tumor infiltration, so a CT-guided biopsy was performed, confirming the diagnosis of sarcomatoid carcinoma. An immunohistochemical panel showed positive staining of tumor cells with pan-cytokeratin (Figure 3).

Following a multidisciplinary consultation meeting, it was decided to initiate corticosteroid therapy, followed by decompression radiotherapy to the encephalon. The potential for palliative chemotherapy was to be reconsidered post-irradiation, contingent upon the patient's overall condition. In the event of clinical improvement, a regimen of weekly carboplatin (AUC2) and paclitaxel would be contemplated. Unfortunately, the patient's condition rapidly deteriorated, resulting in death before treatment could be commenced.

## Discussion

While sarcomatoid carcinoma can arise in various anatomical locations, pulmonary sarcomatoid carcinoma (PSC) remains an uncommon presentation [5]. It represents a rare subset of poorly differentiated non-small cell lung carcinoma (NSCLC), accounting for only 0.1% to 0.4% of all lung cancer cases [11]. PSC typically carries a worse prognosis compared to other NSCLC subtypes [2].

Patients diagnosed with PSC typically present at a median age of 68 years, with tumors averaging a size of 5 cm. They are predominantly male, with a significant history of smoking, reflecting moderate-to-heavy tobacco usage [3]. In our case study, the patient was also male, with an extensive smoking history, although he presented at the age of 55 with a smaller tumor size of 2 cm.

PSC often lacks specific clinical manifestations [5], with patients commonly experiencing symptoms such as dyspnea, cough, hemoptysis, chest pain, and weight loss. These symptoms typically arise approximately one month before clinical assessment and diagnosis, or patients may already manifest complications or metastases, prompting diagnosis [9,10].

The complications associated with PSC mirror those of other lung cancers. Furthermore, metastatic lesions frequently involve multiple organs such as the lungs, adrenal glands, pleura, brain, bones, or liver. Unusual sites of metastasis may include subdiaphragmatic lymph nodes, kidneys, peritoneum, pancreas, skin, and the right ventricle [3]. In our case, the initial symptom was left hemiplegia due to metastasis.

Qin et al.'s study on CT scan diagnosis of PSC highlights several important observations [12]. Peripheral tumors are more common than central ones, often located in the upper lobe. Tumors tend to be large and well-defined, with a round or quasi-circular appearance, though some may exhibit lobulated growth patterns due to varying growth rates. The Burr sign is uncommon (the Burr sign is caused by the thickening of pulmonary interlobular septa, vascular proliferation, and perilesional fibrosis due to inflammation or connective tissue production, and the Burr sign can be seen as short and thin in patients with lung cancer). Plain CT scans typically reveal dense soft tissue within the tumor, sometimes accompanied by extensive patchy necrosis. This uneven necrosis may lead to irregular thick-walled cavities or multiple small, wall-less cavities within necrotic areas. Enhanced CT scans often depict edge-ring-shaped enhancement or uneven patchy enhancement in most masses. Peritoneal masses with local or distant metastases are frequent, and subpleural lesions often invade the chest wall or pleura. Intratumoral calcification is rare.

In summary, while CT scans are instrumental in tumor identification, the definitive diagnosis of PSC hinges on histopathology and immunohistochemistry [3].

A positron emission tomography CT (PET-CT) examination may be warranted in cases of multiple metastases or when the necessity for an enhanced CT scan is unclear [3]. Some studies [13,14] have shown significantly elevated PET uptake in sarcomatoid carcinoma compared to other forms of NSCLC, suggesting PET scans could potentially aid in PSC identification. However, pathological confirmation remains definitive [6]. While PSC can sometimes be diagnosed based on cytologic preparations, obtaining surgical specimens or biopsies via bronchoscopy or CT-guided scanning is necessary for confirmation due to disease heterogeneity [6]. Similarly, in our study, histological evidence was acquired via a CT-guided biopsy due to its inaccessibility by bronchoscopy.

Pathologically, PSC is characterized by the presence of both epithelial and mesenchymal elements within the tumor, distinguishing it from other NSCLC subtypes. Initially described by Virchow in 1865 as a "biphasic" lesion comprising adenocarcinomatous or squamous cell components alongside spindle or giant cell elements forming at least 10% of the tumor mass [15], this description aligns with the current WHO criteria for diagnosing PSC. The disease can be classified into five subgroups: pleomorphic carcinoma (the most prevalent type, accounting for over 50% [16]), spindle cell carcinoma, giant cell carcinoma, carcinosarcoma, and pulmonary blastoma [2].

The spindle and giant cell components of pleomorphic carcinoma often exhibit an epithelial lineage. Markers such as anti-epithelial antigen 1/anti-cytokeratin 3 (AE1/3), cytokeratin-associated membrane protein 5.2 (CAM 5.2), cytokeratin 18 (CK18), and cytokeratin 7 (CK7) are frequently positive, whereas epithelial membrane antigen, carcinoembryonic antigen (CEA), CD15, and Ber-EP4 are less so [17,18]. Positive epithelial markers are not mandatory for diagnosis if components of adenocarcinoma, squamous cell carcinoma, or large cell carcinoma are present. Overexpression of the epidermal growth factor receptor (EGFR), a tyrosine kinase receptor, has been observed in nearly all cases and is implicated in the etiology [19].

Due to its rarity, rapid progression, limited survival, and heterogeneous pathological characteristics, formulating treatment recommendations for PSC has proven challenging [2]. Currently, standard treatments used for conventional NSCLC, including surgery, radiation therapy, and chemotherapy, are employed in managing PSC [20,21]. Complete tumor resection is crucial, especially for patients with localized tumors, as the disease demonstrates resistance to traditional chemotherapy and a limited response to radiotherapy [3]. Surgery, which has shown the most substantial survival benefit in early-stage operable PSC, remains the standard of care for eligible patients [2]. Research is actively exploring alternative treatments to chemotherapy in NSCLC, including the development of molecular-targeted therapies [2]. While the efficacy of targeted therapy in PSC is still under investigation, promising results have been observed with immunotherapy in NSCLC, in conjunction with chemotherapy and targeted therapy [20].

The prognosis for PSC is dismal, even compared to other NSCLC types [2]. Various factors, including histologic type, duration from symptom onset to diagnosis, tumor invasiveness, growth pattern, presence of distant metastases, P stage, extent of surgical resection, vascular/lymphatic invasion, and specific gene mutations such as programmed death-ligand 1 (PD-L1), total lesion glycolysis-primary tumor (TLG-P), and Kirsten rat sarcoma viral oncogene homolog (KRAS) mutations, serve as independent prognostic indicators in PSC [3]. Patients are often diagnosed at intermediate or advanced stages, limiting surgical options [3]. Additionally, PSC's resistance to first-line chemotherapy compounds contributes to its inferior overall survival, which is 20% lower than that of other NSCLC types [22].

## Conclusions

Pulmonary sarcomatoid carcinoma (PSC) is a rare and aggressive subtype of non-small cell lung carcinoma with a poor prognosis and limited treatment options. The case presented underscores the diagnostic and therapeutic challenges associated with PSC, including rapid disease progression and resistance to conventional therapies. Despite advancements in molecular diagnostics and treatment, the prognosis remains bleak. Early detection and a multidisciplinary approach are crucial for managing PSC. Further research is needed to explore targeted therapies and immunotherapies to improve outcomes for patients with this rare and deadly disease.
